# Targeted Isolation of Tsitsikammamines from the Antarctic Deep-Sea Sponge *Latrunculia biformis* by Molecular Networking and Anticancer Activity

**DOI:** 10.3390/md16080268

**Published:** 2018-08-02

**Authors:** Fengjie Li, Dorte Janussen, Christian Peifer, Ignacio Pérez-Victoria, Deniz Tasdemir

**Affiliations:** 1 GEOMAR Centre for Marine Biotechnology (GEOMAR-Biotech), Research Unit Marine Natural Products Chemistry, GEOMAR Helmholtz Centre for Ocean Research Kiel, Am Kiel-Kanal 44, 24106 Kiel, Germany; fli@geomar.de; 2 Senckenberg Research Institute and Natural History Museum, Senckenberganlage 25, 60325 Frankfurt, Germany; dorte.janussen@senckenberg.de; 3 Pharmaceutical Chemistry, Kiel University, Gutenbergstraße 76, 24118 Kiel, Germany; cpeifer@pharmazie.uni-kiel.de; 4 Fundación MEDINA, Parque Tecnológico de la Salud, Av. Conocimiento 18016 Granada, Spain; ignacio.perez-victoria@medinaandalucia.es; 5 Faculty of Mathematics and Natural Sciences, Kiel University, Christian-Albrechts-Platz 4, 24118 Kiel, Germany

**Keywords:** Antarctica, deep-sea, marine sponge, *Latrunculia*, molecular networking, molecular docking, tsitsikammamine

## Abstract

The Antarctic deep-sea sponge *Latrunculia* (*Latrunculia*) *biformis* Kirkpatrick, 1908 (Class Demospongiae Sollas, Order Poecilosclerida Topsent, Latrunculiidae Topsent) was selected for chemical analyses due to its potent anticancer activity. Metabolomic analysis of its crude extract by HRMS/MS-based molecular networking showed the presence of several clusters of pyrroloiminoquinone alkaloids, i.e., discorhabdin and epinardin-type brominated pyridopyrroloquinolines and tsitsikammamines, the non-brominated bis-pyrroloiminoquinones. Molecular networking approach combined with a bioactivity-guided isolation led to the targeted isolation of the known pyrroloiminoquinone tsitsikammamine A (**1**) and its new analog 16,17-dehydrotsitsikammamine A (**2**). The chemical structures of the compounds **1** and **2** were elucidated by spectroscopic analysis (one-dimensional (1D) and two-dimensional (2D) NMR, HR-ESIMS). Due to minute amounts, molecular modeling and docking was used to assess potential affinities to potential targets of the isolated compounds, including DNA intercalation, topoisomerase I-II, and indoleamine 2,3-dioxygenase enzymes. Tsitsikammamines represent a small class of pyrroloiminoquinone alkaloids that have only previously been reported from the South African sponge genus *Tsitsikamma* Samaai & Kelly and an Australian species of the sponge genus *Zyzzya* de Laubenfels. This is the first report of tsitsikammamines from the genus *Latrunculia* du Bocage and the successful application of molecular networking in the identification of comprehensive chemical inventory of *L.* *biformis* followed by targeted isolation of new molecules. This study highlights the high productivity of secondary metabolites of *Latrunculia* sponges and may shed new light on their biosynthetic origin and chemotaxonomy.

## 1. Introduction

Marine sponges are the richest and best-studied sources of bioactive marine natural products (MNPs) among all marine organisms [1]. Sponges contribute to nearly 30% of all MNPs [2,3]. Antarctica is considered as one of the most hostile environments on earth, with extremely cold temperatures, long dark/light periods and low nutrients levels [4]. At the same time, it is one of the oldest, environmentally stable and well-structured marine systems with a rich benthic community [5]. Sponges are often highly diverse and dominant in the invertebrate communities of Antarctic ecosystems [6,7,8]. They provide structure to the benthos and nursery grounds for juvenile fish and many other organisms [9]. In order to cope with harsh environmental conditions, to deter large predators such as sea stars and nudibranchs, to avoid fouling and to ensure their survival in Antarctica, sponges have developed a range of behavioral, physical and chemical defense mechanisms [6,10] including the production of ‘antifreeze’ peptides [11] and unique, highly complex secondary metabolites [12,13]. While the majority of new therapeutic agents have been, and are sourced from tropical or temperate oceans, Antarctica and the polar regions generally, are largely unknown from this standpoint [14,15], due to issues of access.

Until recently, the sponge family Latrunculidae Topsent (Class Demospongiae Sollas, Order Poecilosclerida Topsent) comprised five key genera: *Latrunculia* du Bocage, *Strongylodesma* Lévi, Sceptrella Schmidt, *Tsitsikamma* Samaai & Kelly and *Cyclacanthia* Samaai, Govender & Kelly [16,17,18]; *Bomba* Kelly, Reiswig & Samaai and *Latrunclava* Kelly, Reiswig & Samaai were recently discovered in the North Pacific [19]. From a thorough taxonomic re-evaluation of the various genera in which pyroloquinolines have been found and of other compounds attributed to Latrunculiidae genera [20], definitive new taxonomic conclusions were able to be drawn, resulting in the greater integrity of Latrunculiidae (separating it from Podospongiidae), and the establishment of new genera within Latrunculiidae [18,20].

Various subclasses of pyrroloiminoquinone alkaloids are represented in latrunculid sponges. *Latrunculia* species are predominantly distributed in cold-water regions along the coasts and on the continental shelfs of New Zealand, South Africa, Antarctica, and the North Pacific [17,18,19]. Since the first report of discorhabdin C, a brominated pyridopyrroloquinoline alkaloid from a New Zealand *Latrunculia* sp. in 1986 [21], this genus has emerged as a prolific source of discorhabdins [22,23,24,25]. The core tetracyclic iminoquinone chromophore renders discorhabdins and many other pyrroloiminoquinone alkaloids highly coloured, producing the diagnostic deep emerald and olive greens, and brick red coloration of many Latrunculia sponges in life [26]. Discorhabdin pigments have been shown to be located in the outer layers of the Antarctic sponge species *Latrunculia* (*Latrunculia*) *apicalis* Ridley & Dendy, and linked to antifouling activity and the chemical defense of the sponge against the spongivorous sea star *Perknstar fuscus* [27]. Besides discorhabdins, the genus *Latrunculia* has repeatedly been reported to contain makaluvamines (pyrroloiminoquinones), epinardins (pyridopyrroloquinolines) and makaluvic acids (pyrroloquinoline) [28,29]. Tsitsikammamines that possess a non-brominated bis-pyrroloiminoquinone scaffold represent another group of pyrroloiminoquinone alkaloids that are closely related to discorhabdins. This small alkaloid family has only been reported from the South African latrunculid sponge *Tsitsikamma favus* [28,30] and an Australian *Zyzzya* sp. [31]. Tsitsikammamines have previously been suggested to represent biosynthetic intermediates between discorhabdins and makaluvamines, and as chemotaxonomic markers for the genus *Tsitsikamma* [28,30]. A potential microbial origin of tsitsikammamines is currently being debated [32,33]. Discorhabdins exhibit strong anticancer activities by inhibiting the interaction of the cancer target HIF-1*α* (hypoxia-inducible factor 1alpha) with p300 [34]. The anticancer activity of the natural tsitsikammamines has been linked to DNA intercalation and inhibition of topoisomerase I and II enzymes [28], while synthetically prepared tsitsikammamines and derivatives have been shown to inhibit indoleamine 2,3-dioxygenase (TDO1) and tryptophan 2,3-dioxygenase (TDO) enzymes [35,36].

As part of our recent project involving chemical and biological investigations of Antarctic deep-sea sponges, the crude extract of *L. biformis*, which was collected from the Antarctic Weddell Sea shelf at -303 m depth was found to show significant anticancer activity against six cancer cell lines. A comprehensive dereplication study employing HRMS/MS-based molecular networking (MN) allowed the identification of several molecular clusters in the crude extract, including a large cluster composed of discorhabdins and epinardins, as well as a small tsitsikammamine cluster. By using a combination of MN and anticancer activity-guided isolation, we obtained two minor tsitsikammamine-type alkaloids, namely the known compound tsitsikammamine A (**1**) and its new dehydro-analog 16,17-dehydrotsitsikammamine A (**2**) from *L. biformis*. Their structures were elucidated on the basis of high-resolution spectroscopic methods (NMR and HR-ESIMS) and by comparison with data reported in the literature. Since the amounts of the pure compounds were very minor excluding any possibility for bioactivity testing, we used a structure based docking approach against several cancer targets reported for **1,** namely DNA intercalation, topoisomerase I-II, and indoleamine 2,3-dioxygenase (IDO1) enzymes, to predict anticancer potential of **1** and **2**. Docking represents an established method in drug discovery projects enabling insights into molecular ligand-protein interactions. This is the first report of tsitsikammamine type alkaloids from a species of *Latrunculia* and highly the successful application of MN in metabolome analysis and targeted isolation of new molecules from a complex sponge extract.

## 2. Results

### 2.1. Molecular Networking-Based Dereplication

The dark green-colored sponge material was freeze-dried and successively extracted with water, MeOH and DCM. The organic extracts were combined and submitted to bioactivity screening against six cancer cell lines. The high anticancer activity of the crude extract (IC_50_ values from 0.8 to 6.3 µg/mL, Table 1) prompted us to undertake a detailed chemical profiling on the crude sponge extract.

In order to achieve an in-depth dereplication of the known compounds and to prioritize the isolation workflow towards putatively undescribed molecules, the crude extract of *L. biformis* was analysed by tandem UPLC-QToF-MS/MS (positive-ion mode). The MS/MS (MS^2^) data were uploaded to the publicly available Global Natural Product Social molecular networking (GNPS) platform (http://gnps.ucsd.edu) and analysed while using the molecular networking (MN) online workflow [37]. Molecular networking is a bioinformatics tool for dereplication of complex metabolomes, providing visual displays of the chemical space present in MS^2^ experiments. The visualization approach by a special software allows detection and grouping of sets of MS^2^ spectra from molecules (the so-called spectral networks), which have similar structures with similar fragmentation patterns. In MN visualization, each MS^2^ spectrum is represented as a node (compound) whereas spectrum-to-spectrum alignments are shown as edges (connections) between nodes. The thicker the edge, the higher the similarity between the nodes [38].

**Figure 1:**
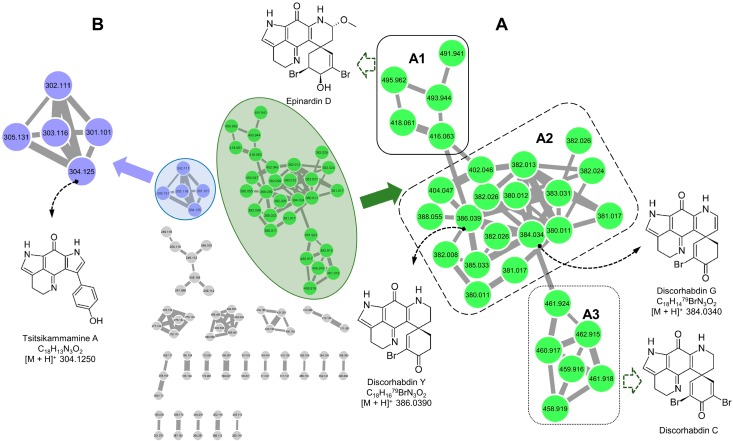
Annotated molecular network of the *L. biformis* extract. Numbers within the nodes indicate parent ions, and edge thickness represents the cosine similarity between nodes. Green nodes represent discorhabdins and epinardins. Purple nodes represent tsitsikammamines. (**A**) Discorhabdin and epinardin cluster. (**A1**) Epinardin sub-cluster. (**A2**) Monobrominated discorhabdin subcluster. (**A3**) Dibrominated discorhabdin sub-cluster. (**B**) Tsitsikammamine cluster.

The composite MN of the crude *L. biformis* extract consisted overall of 90 nodes, which were grouped into 22 clusters (at least two nodes per cluster) after subtraction of the nodes from the solvent control (Figure 1). Some nodes represented isotopes or adducts, thus not all network nodes correspond to a single molecule. Initially, the automated dereplication by comparison of MS^2^ fragmentation profiles with reference spectra from 25 spectral libraries on GNPS did not annotate any compounds. This is not surprising because GNPS contains predominantly microbial metabolites and the availability of data on sponge metabolites is limited in the GNPS spectral library. Hence after the generation of the MN, a manual dereplication was performed by searching the predicted molecular formulae of these nodes against several databases, such as Dictionary of Natural Products, MarinLit, Reaxys and SciFinder. The Competitive Fragmentation Modeling for Metabolite Identification (CFM-ID) platform (http://cfmid.wishartlab.com) was used to predict the fragments of a molecule and to distinguish compounds with the same molecular formula. Taking the elemental composition, biological source and the fragmentation pattern into consideration, this manual dereplication method allowed the identification of three known pyrroloiminoquinone alkaloids discorhabdins G, Y, and tsitsikammamine A (Figure 1).

A detailed examination of the MN permitted annotation of the two main molecular families (Figure 1). With 29 nodes, the first discorhabdin family (cluster A) was the biggest cluster with all of the compounds being brominated. A detailed examination indicated this cluster to comprise of three subfamilies (A1–A3) separated based on the number of bromine atoms and methoxy substitution (Figure 1). The largest subfamily A2 contained only mono-brominated discorhabdins. In a database search, the molecular formula of one node at *m/z* 384.0340 (C_18_H_15_^79^BrN_3_O_2_, [M + H]^+^) corresponded to two molecules, discorhabdin E and G. Based on key fragments *m/z* 367.0300 (C_18_H_12_^79^BrN_2_O_2_), 357.0400 (C_17_H_14_^79^BrN_2_O_2_) and 304.1300 (C_18_H_14_N_3_O_2_) obtained by prediction on CFM-ID platform, this node was rapidly annotated as discorhabdin G (Figure 1). Another node at *m/z* 386.0390, ([M + H]^+^, C_18_H_17_^79^BrN_3_O_2_) also yielded two hits (discorhabdin Y and V) in the database search. It was subsequently identified as discorhabdin Y based on the predicted MS fragments at *m/z* 369.0200 (C_18_H_14_^79^BrN_2_O_2_), 359.0200 (C_17_H_16_^79^BrN_2_O_2_), 306.1400 (C_18_H_16_N_3_O_2_) and 277.1400 (C_17_H_13_N_2_O_2_). In addition, seven nodes were connected to discorhabdin G and eight nodes were linked to discorhabdin Y in the subfamily A2. However, none of these chemical formulae matched to any known compound in the databases, indicating them to be potentially new discorhabdin derivatives.

The subfamily A1 (Figure 1) contained five nodes and it was identified as an epinardin subcluster, separated from the subfamily A2 by bearing two bromine atoms and a methoxy function. Two nodes with *m/z* [M + H]^+^ 416.0630 (C_19_H_19_^79^BrN_3_O_3_) and *m/z* [M + H]^+^ 491.9410 (C_19_H_16_^79^Br_2_N_3_O_3_) from this subfamily had resulted in no hits in MarinLit, but based on similarity of their MS^2^ fragmentation pattern on CFM-ID platform, these two nodes were identified as new derivatives of epinardin D *m/z* 494.9793 (C_19_H_19_^79^Br_2_N_3_O_3_) [39]. The node at *m/z* 416.0630 [M + H]^+^ was connected to two nodes in subfamily A2, indicating its structural similarity to the discorhabdin subcluster. The other three nodes in A1 were annotated as bromine isotope ions of these two compounds.

The subfamily A3 (Figure 1) was comprised of six nodes. One node with *m/z* [M + H]^+^ 459.9160 (C_18_H_12_^79^Br_2_N_3_O_2_) gave no match in MarinLit but the high similarity of its MS^2^ spectrum with that of discorhabdin C (generated on CFM-ID) indicated it to be a new derivative of discorhabdin C (*m/z* 460.9374, C_18_H_13_^79^Br_2_N_3_O_2_) [21]. The other five nodes in subcluster A3 were annotated as isotope ions of this molecule. The second bromine atom in the subcluster A3 separated it from the monobrominated discorhabdins in subfamily A2.

The second cluster (B) included five nodes, one of which with *m/z* 304.1250 (C_18_H_14_N_3_O_2_ [M + H]^+^) was annotated as tsitsikammamine A [30]. This finding was unexpected because tsitsikammamines have never previously been reported from the genus *Latrunculia*. In this cluster, we noted one node with *m/z* 302.1110 (C_18_H_12_N_3_O_2_, [M + H]^+^), which was connected to that of tsitsikammamine A with a very thick edge indicating a high structural similarity. The absence of a molecular formula match to any known compound in the databases indicated this compound to be a putatively new analog of tsitsikammamine A. The other nodes in cluster B were annotated as the minute isotope ions of tsitsikammamine A and its new analog. There was no bromination in this cluster.

The remaining clusters (shown as grey networks in Figure 1) remained unidentified as they had no match or MS^2^ fragment similarity to any known compound in multiple commercial or publicly available databases. Hence they potentially represent new clusters and compounds. Interestingly, no makaluvamine type of compounds were detected in the crude extract.

### 2.2. Purification and Structure Elucidation

The crude organic extract of the sponge that showed high activity against all six human cancer cell lines (Table 1) was fractionated over an SPE cartridge to yield five main fractions. Fraction 3 (44 mg) that retained the anticancer activity (IC_50_ values ranging from 1.2 to 5.6 µg/mL, Table 1) of the crude extract and contained tsitsikammamines, discorhabdins and epinardins as determined by LC-MS analyses, was given priority for chemical work-up. RP-HPLC separation of the fraction 3 yielded 33 sub-fractions. Tsitsikammamines were tracked in sub-fraction 8 (1.3 mg) while discorhabdins and epinardins were tracked in sub-fraction 9 (2.4 mg). Further RP-HPLC purification of sub-fraction 8 yielded compounds **1** and **2** in very minor amounts. Although the remaining sub-fraction 9 had a slightly higher amount, it was not worked up, due to the much higher diversity of discorhabdins and epinardins.

**Figure 2:**
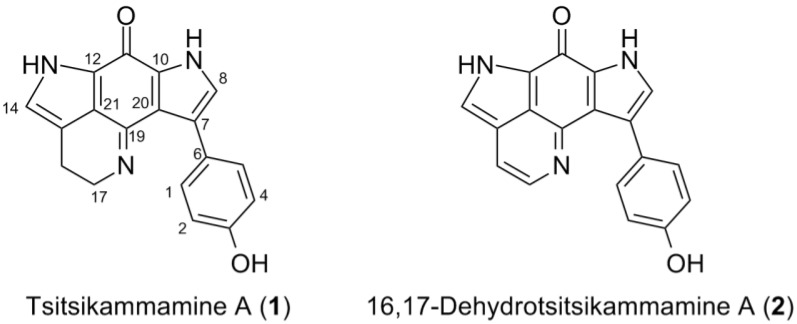
Chemical structures of compounds **1** and **2**.

**Figure 3:**
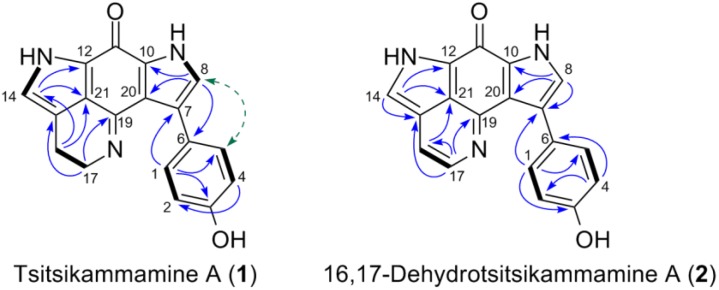
Key two-dimensional (2D) NMR correlations observed for compounds **1** and **2**. The COSY (in bold), key H→C HMBC (arrows), and H→H NOE (dashed line).

The compound **1** was obtained as a greenish film. Its molecular formula C_18_H_13_N_3_O_2_ was deduced by HR-ESIMS (*m/z* 304.1110 [M + H]^+^) indicating 14 degrees of unsaturation. The ^1^H NMR spectrum in DMSO-*d*_6_ contained an AB system at *δ*_H_ 7.71 (2H, d, *J* = 8.5 Hz) and *δ*_H_ 6.70 (2H, d, *J* = 8.5 Hz) indicating the presence of a *para*-disubstituted benzene ring, two aromatic resonances at *δ*_H_ 7.14 (1H, d, *J* = 1.8 Hz) and *δ*_H_ 6.91 (1H, s), two triplets at *δ*_H_ 3.97 (2H, t, *J* = 7.8 Hz) and *δ*_H_ 2.65 (2H, t, *J* = 7.8 Hz), plus three exchangeable protons (*δ*_H_ 9.31, 12.00 and 12.28, all s) (Table 2, Figure S1). The ^13^C NMR spectrum showed 16 carbon signals including two methylenes (*δ*_C_ 17.9 and *δ*_C_ 49.7), four aromatic methines (*δ*_C_ 114.4, 121.2, 122.8, 129.8), nine quaternary carbons (*δ*_C_ 117.4, 121.6, 121.8, 124.3, 124.7, 125.1, 133.2, 154.1, 156.1) and a carbonyl group at *δ*_C_ 167.7 (Table 3, Figure S2). A detailed analysis of the one-dimensional (1D) NMR data of **1** revealed high similarity to those reported for tsitsikammamine A (Figure 2), a nonbrominated bis-pyrroloiminoquinone alkaloid previously reported from the South African sponge *Tsitsikamma favus* [28,30]. The main differences that were observed between the two compounds stemmed from the chemical shifts of the region C12 through C20 and were attributed to the fact that compound **1** was isolated as a free base whereas tsitsikammamine A was obtained as an N-18 TFA salt [28,30]. The addition of one drop of TFA rapidly converted **1** into its TFA salt, upon which all ^1^H and ^13^C resonances became superimposable with those reported for tsitsikammamine A (Table 2 and Table 3; Figures S8 and S9). The only exception was the NMR data of the *para*-disubstituted benzene ring that needed revision. Based on a strong NOESY correlation with H-8 (*δ*_H_ 7.14, d, *J* = 1.8), two equal aromatic protons (*δ*_H_ 7.71) were assigned to H-1/H-5, which was ascribed *vice versa* (as H-2/H-4) in the literature [30]. A full set of two-dimensional (2D) NMR experiments was run to unambiguously confirm the tsitsikammamine scaffold. The key HMBC and NOESY correlations observed are shown in Figure 3, Table 2 and Table 3 display the complete NMR data of **1**, as free-base and TFA salt in both DMSO-*d*_6_ and CD_3_OD. Hence compound **1** was unambiguously characterized as tsitsikammamine A. 

The compound **2** was also purified as a greenish film. The molecular formula C_18_H_11_N_3_O_2_ assigned to **2** by HR-ESIMS (*m/z* 302.0911 [M + H]^+^) indicated the presence of 15 degrees of unsaturation. Comparison of the ^1^H-NMR data of **2** with those of compound **1** (Table 2) revealed a high resemblance, with the only difference being the replacement of the methylene signals (H-16 *δ*_H_ 2.89 and H-17 *δ*_H_ 3.94) with two olefinic signals (H-16, *δ*_H_ 7.66, d, *J* = 5.9 Hz and H-17, *δ*_H_ 8.34, d, *J* = 5.9 Hz) indicating an unsaturation in **2**. The key HMBC correlations (Figure 3) between H-16 and C-21 (*δ*_C_ 120.7); H-17 and C-15 (*δ*_C_ 124.8), C-16 (*δ*_C_ 114.0) and C-19 (*δ*_C_ 146.7) indicated the position of the additional double bond at ∆^16^. The strong COSY correlations between H-16 and H-17 confirmed this assignment. Our attempts to locate the carbonyl carbon (C-11) using high-resolution cryoprobe or 1.7 mm microcryoprobe NMR was unsuccessful, however, the structural homology was obvious from the ^1^H NMR and 2D NMR data. Finally, the two mass unit difference between the molecular formulae of **1** and **2**, as determined by HR-ESIMS data, clearly supported **2** being the ∆^16^ unsaturated analog of **1**. Thus the chemical structure of **2** was identified as 16,17-dehydrotsitsikammamine A (Figure 2). Compound **2** represents the node that was identified above as a putatively new analog of tsitsikammamine A by molecular network (cluster B, Figure 1).

### 2.3. Molecular Modeling and Docking

Both tsitsikammamines and discorhabdins have been reported to show potent anticancer activities in vitro [28,30,40]. On a molecular level, DNA intercalation, topoisomerase I and II, indoleamine 2,3-dioxygenase (IDO1) and tryptophan 2,3-dioxygenase (TDO) enzymes have been identified as potential targets for tsitsikammamines or their synthetic derivatives, while the inhibition of HIF-1α/p300 interaction has been reported as potential target of discorhabdins [28,34,35,36]. Because both compounds **1** and **2** were isolated in very little amounts, we were unable to test them against any cancer cell lines or pursue any mechanistic studies with any of these enzymes. Hence, in order to assess the potential anticancer activity of our compounds, we opted for a virtual approach by molecular modeling (using Schrödinger software Maestro; www.schrodinger.com). Where possible, based on suitable pdb structures, we performed docking experiments of compounds tsitsikammamine A (**1**) and 16,17-dehydrotsitsikammamine A (**2**) within the binding sites of their reported possible targets (topoisomerase I/II, indoleamine 2,3-dioxygenase IDO1). Thus, we prepared available relevant pdb protein structures, removed the original ligands and generated receptor grids. Small molecule three-dimensional (3D) structures of **1** and **2** were energetically minimized and possible tautomers/protonated states were evaluated (LigPrep) to yield one compound structure each. Using these optimized ligand structures, docking into the prepared respective active site was performed by Glide XP [41]. Calculated 3D binding modes were illustrated, or presented as 2D ligand-interaction diagrams, for clarity.

**Figure 4:**
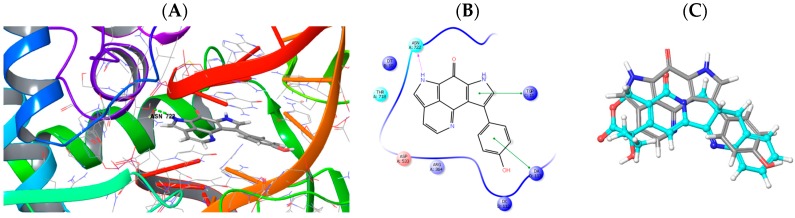
(**A**) Calculated three-dimensional (3D) binding mode of **2** in the crystallographically determined active site of topoisomerase I (pdb 1T8I) also containing a DNA molecule (coloured in red) with a single strand break. (**B**) Corresponding 2D ligand interaction diagram showing key interactions of **2** to topoisomerase I and DNA. (**C**) Overlay of the binding pose of **2** (grey) with the original ligand camptothecin (turquoise) indicating similar ligand space occupied by the scaffolds.

Docking of compound **2** into the active site of topoisomerase I (pdb 1T8I) [42] yielded plausible binding modes (Figure 4). When compared to the original ligand camptothecin, the flat aromatic core of **2** intercalates into the DNA part forming aromatic π-π-stacking interactions while addressing an H-bond towards ASN 722 of the topoisomerase I protein. A similar binding pose was calculated for **1** in which the 16,17-dehydrotsitsikammamine scaffold is slightly distorted compared to **2** (not shown). Similar results were obtained by docking experiments with topoisomerase II (pdb 3QX3, original ligand etoposide) [43], likely indicating these proteins to be targets for marine derived tsitsikammamines **1** and **2**.

**Figure 5:**
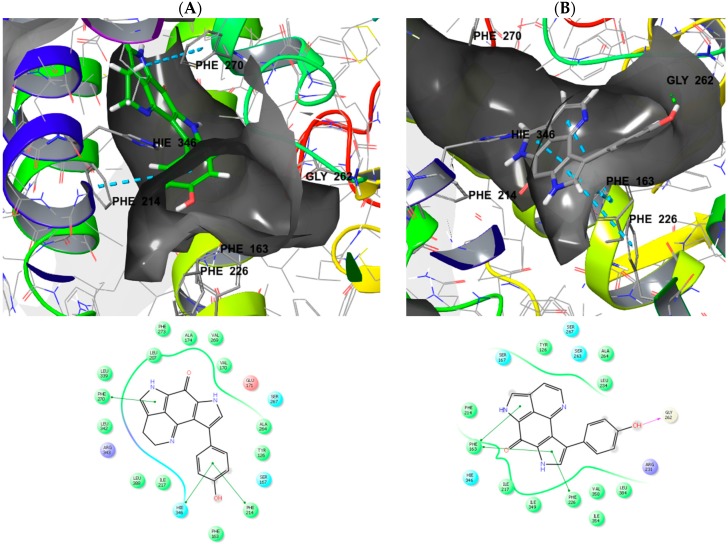
3D binding poses and ligand interaction diagrams of **1** (**A**, green) and **2** (**B**, grey) in the crystallographically determined active site of indoleamine 2,3-dioxygenase (IDO1) (pdb 6AZW) with key interactions. The binding pocket is shown in a similar orientation, respectively. Ligand docking revealed two different binding poses with **1** buried deeper in the pocket when compared to **2**. Noteworthy, similar results were obtained by docking of **1** and **2** in IDO1 structure pdb 6AZV (not shown).

We next focussed on docking experiments with another reported target for tsitsikammamines, namely the enzyme indoleamine 2,3-dioxygenase (IDO1) [36] for which valid structural data including ligand-protein complexes is also available. As the cofactor to mediate substrate oxidation IDO1 contains a heme moiety and ligands typically form interactions by complexing the Fe central atom. Examples for such IDO1 inhibitors and relevant interacting moieties include NLG919 derivative (imidazole-like nitrogen in pdb 5EK2) or ligand INCB14943 (hydroxylamidine moiety in pdb 5XE1). Since **1** and **2** do not bear comparable nitrogen functionalities to be able to interact with heme in a similar manner, docking of the compounds into these active sites revealed no plausible binding modes. However, recent reports demonstrated that another class of potent IDO1 inhibitors such as FXB-001116 (pdb 6AZW) and BMS-978587 (pdb 6AZV) [44] bind to the IDO1 apo structure with high affinity, thereby displacing the heme moiety. Accordingly, docking of **1** and **2** suggested possible binding modes in the apo active sites of IDO1 (Figure 5) suggesting both compounds to be apo-IDO1 ligands.

Inspecting the docking poses of tsitsikammamine A (**1**) and its derivative 16,17-dehydrotsitsikammamine A (**2**) in the above mentioned protein structures on a molecular level suggests slight evidence for the role of unsaturation. Compound **2** shows a totally planar aromatic core, which is somewhat conformationally twisted out of plane in the case of **1** having two sp3 carbon atoms in the ethyl bridge. Thus, the total planar core of **2** preventing the ligand to access deeper into the IDO1 binding pocket, addressing aromatic interactions towards front residues Phe163/Phe226, and additionally forming an H-bond towards GLY262 within the solvent opening area. In contrast, **1** is actually filling the deep lipophilic pocket of IDO1, thereby not able to address an H-bond to solvent accessible residues. Interestingly, when compared to **2**, compound **1** is showing an about 180° twisted core dominated by aromatic interactions to side chain residues Phe214/Phe270/Hie346. However, structure-activity relationship studies (SARs) clearly pointed out the diminished cytotoxicity in discorhabdins with ∆^16^ unsaturation [45,46]. The debated double bond of **2** is not part of an electrophilic reactive α,β-unsaturated Michael acceptor system (“warhead”), which could covalently attach to the binding site. While our molecular modeling approach suggested possible binding interactions for **1** and **2** towards topoisomerases/DNA intercalation and IDO1, structural data for the further possible targets such as tryptophan 2,3-dioxygenase (TDO) and HIF-1α/p300 protein was not sufficient to draw analogous conclusions.

To our knowledge, this is the first molecular docking study performed on non-synthetic tsitsikammamines. Based on the literature reports [28,30] and the activity profile of the crude extract and the fraction 3 from which compounds **1**–**2** were isolated (Table 1, Figure S18), it is reasonable to expect strong anticancer activity from both compounds. However, the IC_50_ values against the non-cancerous human cell line (HaCaT) indicate that anticancer activity is associated with general toxicity, which is common for pyrroloiminoquinone alkaloids [28]. Nevertheless, almost eight times higher potency of fraction 3 against the human colon cancer cell line (HCT-116) points out to some degree of selectivity.

## 3. Discussion

The identification and isolation of tsitsikammamines from a species of *Latrunculia* raises questions regarding the origin of the tsitsikammamines. It should be noted that, the possibility of a misidentification of the study material is rejected by the discovery of the unique spiculation of the specimen under study, and is supported by the fact that the genus *Tsitsikamma* is endemic to South Africa [32] and has not been reported from other Pacific, Atlantic or polar regions [19] that harbour latrunculid sponges. It is also unlikely that the tsitsikammines are present, but undetectable, at a broader taxonomic level of order (Poecilosclerida) or family (Latrunculiidae, Acarnidae) as the Latrunculiidae, Acarnidae (Zyzzya most particularly), and Podospongiidae (previously integrated into Latrunculiidae) are now amongst the earliest and best known of groups in the search for MNPs [18,20].

There are current debates on the natural origin of discorhabdins and tsitsikammamines. An early biosynthetic study carried out by the group of Blunt and Munro [47] showed that discorhabdin B, the principal cytotoxic pigment of a New Zealand *Latrunculia* sp., is of sponge origin, discounting a microbial source for discorhabdins. In contrast, tsitsikammamines have been hypothesized to have a microbial origin, and the sponge produced these compounds, *T. favus*, has been shown to contain unusual bacterial taxa dominated by a novel *Betaproteobacterium* sp. [32]. A recent study by Matcher et al. (2016) showed that several sponges belonging to genera *Latrunculia*, *Tsitsikamma* and *Cyclacanthia* were dominated by the same *Betaproteobacterium* sp., suggesting that this bacterium may have co-evolved with their latrunculid sponge hosts [33]. The *Tsitsikamma* and *Cyclacanthia* sp. also contain a Spirochaetae OUT that was possibly acquired by the sponges recently from their environments [33]. To our knowledge, there is no report on the symbionts of *Zyzzya* sp., although tsitsikammamine C was discovered in an Australian *Zyzzya* sponge [31]. Hence the question regarding the true natural origin of tsitsikammamines remains open.

So far only five tsitsikammamines are known in the literature, i.e., tsitsikammamines A, B, C and the N-18 oximes of tsitsikammamine A and B. The N-18 oximes have been suggested as artifacts of tsitsikammamines A, B formed during the isolation process [28]. Taboada et al. (2010) have briefly mentioned the presence of tsitsikammamine A in *Latrunculia brevis* in 2010 [48], but this information was cited as unpublished data without a subsequent publication. So by now, tsitsikammamine A, B, and their N-18 oximes have only been ‘officially’ reported from one single South African *Tsitsikamma* sp., *T. favus* and exhibit anticancer activity [28]. Tsitsikammamine C that was reported from an Australian *Zyzzya* sponge [31] exerts antimalarial activity. The current study adds 16,17-dehydrotsitsikammamine A (**2**), as the sixth member of this small family with potential anticancer activity as determined by molecular docking studies with enzyme targets previously reported for tsitsikammamines.

MS/MS-based molecular networking is gaining enormous popularity among the natural product community, enabling efficient metabolomics and dereplication of molecular clusters in complex extracts and fractions. This computer-based approach is highly advantageous and superior to classical metabolomics, because instead of identifying single metabolites, it characterizes and groups large number of related new, known or new derivatives of known family of compounds in multiple molecular families (networks) giving clues on exhaustive chemical inventory of a biological organism. MN is a versatile method that has been used in many areas including drug discovery, ecology, microbiology, and biosynthesis [38,49,50]. The current study confirms that MN has an outstanding potential in chemotaxonomical studies by efficiently mapping the chemical space of a biological organism and clearly clustering highly related subfamilies of the pyrroloiminoquinone scaffold by skeletal features (e.g., tsitsikammamines as non-brominated bis-pyrroloiminoquinones) and substitution patterns (discorhabdins and epinardins with mono-/di-bromination, or methoxy substutition). Although only a small amount of sponge biomass was available as a starting material, HRMS^2^-based MN allowed for us to distinguish, group and accurately visualize these subfamilies based on their structural similarities. Furthermore, it enabled rapid and targeted isolation of tsitsikammamines from the *Latrunculia* sponge. The MN showed that *L. biformis* extract contains many new discorhabdins and epinardins (subclusters A1–A3) and even additional molecular clusters (shown in grey colours in Figure 1) that could not be annotated to any scaffold. Hence they may potentially be new classes of natural products.

The taxonomy and systematics of the family Latrunculiidae has been complex and controversial [18,51,52], and the arrangements in the family are still being refined [19]. However, *L. biformis* is easily recognisable due to the possession of an additional form of anisodiscorhabd with a long apical spine, and it has been described from different sites around Antarctica and the Shetland islands [17,53]. Only a single report on the fatty acid composition of this species is known [54]; to our knowledge, this is the first study investigating the rich pyrroloiminoquinone content of this species. The presence of tsitsikammamines, in addition to discorhabdins and epinardins in *L. biformis*, indicates very close and complex biosynthetic relationships of the pyrroloiminoquinone alkaloids among latrunculid sponges, warranting in-depth chemical, biosynthetic and microbiological studies. Following our discovery and structural elucidation, solid SAR studies for **1** and **2** will reveal their potential as pharmacologically relevant inhibitors.

## 4. Materials and Methods

### 4.1. General Procedures

NMR spectra were obtained on a Bruker AV 600 spectrometer (600 and 150 MHz for ^1^H and ^13^C NMR, respectively, Bruker^®^, Billerica, MA, USA) equipped with 5.0 mm Shigemi tube (SHIGEMI, Co., LTD., Tokyo, Japan) or a Bruker Avance III spectrometer (500 and 125 MHz for ^1^H and ^13^C NMR, respectively, Bruker^®^, Billerica, MA, USA) equipped with a 1.7 mm TCI MicroCryoProbe (Bruker^®^, Billerica, MA, USA). The residual solvent signals were used as internal references: *δ*_H_ 3.31/*δ*_C_ 49.0 ppm (MeOD), and *δ*_H_ 2.50/*δ*_C_ 39.51 ppm (DMSO-*d*_6_). 4-Dimethyl-4-silapentane-1-sulfonic acid (DSS) served as the internal standard. High-resolution MS^2^ data were recorded on a Waters Xevo G2-XS QTof Mass Spectrometer (Waters^®^, Milford, MA, USA) coupled to a Waters Acquity I-Class UPLC system (Waters^®^, Milford, MA, USA). HR-ESIMS was recorded on micrOTOF II-High-performance TOF-MS system (Bruker^®^, Billerica, MA, USA) equipped with an electrospray ionization source. Solid phase extraction (SPE) was performed on the Chromabond SPE C18 column cartridges (6 mL/2000 mg, Macherey-Nagel, Duren, Germany). HPLC separations were performed on a VWR Hitachi Chromaster system (VWR International, Allison Park, PA, USA) consisting of a 5430 diode array detector (VWR International, Allison Park, PA, USA), a 5310 column oven, a 5260 autosampler and a 5110 pump combined in parallel with a VWR Evaporative Light Scattering Detector (ELSD 90, VWR International, Allison Park, PA, USA). The eluents that were used for HPLC separations were 20 mM NH_4_CHOO water solution (A) and MeCN (B). Routine HPLC separations were performed on semipreparative (Onyx, 100 × 10 mm, Phenomenex, Torrance, CA, USA) C18 Monolithic column and analytic (SeQuant^®^, 250 × 4.6 mm, Merk, Germany) ZIC-HILIC column. The organic solvents that were used for UPLC-QToF-MS/MS analyses were ULC/MS grade (Biosolve BV, Valkenswaard, The Netherlands) and HPLC grade (ITW Reagents, Germany) for HPLC isolation processes. The water used was MilliQ-water produced by Arium^®^ Water Purification Systems (Sartorius, Germany).

### 4.2. Sponge Material

The green sponge was collected by Daniel Kersken (Senckenberg Research Institute and Natural History Museum), with an Agassiz trawl at a depth of 303 m in the Weddel Sea, Antarctica (70.892° S-11.130° W) on December 2015, during the scientific expedition PS96 (RV POLARSTERN). Taxonomic examination of the spicule complement and dimensions revealed two forms of anisodiscorhabd spicules, one of which has a well-developed spined apical projection, and which is diagnostic for the well-known Antarctic and Southern Ocean sponge species [53], Latrunculia (Latrunculia) biformis Kirkpatrick, 1908 (Class Demospongiae Sollas, Order Poecilosclerida Topsent, Latrunculiidae Topsent) [55]. A reference specimen has been deposited at Senckenberg Research Institute and Natural History Museum, Germany, under the accessing number SMF 12106.

### 4.3. Extraction and Isolation

The sponge tissue (19.141 g, frozen weight) was cut into small pieces and then dried using a freeze-drier (Martin Christ, Germany). The lyophilized biomass (4.651 g) was extracted at room temperature with water (3 × 200 mL) under agitation to yield the aqueous extract (E1, 1.590 g). The sponge residue (3.019 g, dry weight) was subsequently extracted with MeOH (3 × 150 mL), and then with dichloromethane (DCM, 3 × 150 mL) under the same conditions. The MeOH and DCM extracts were combined and evaporated to dryness by a rotary evaporator to yield the organic extract (172 mg). The organic extract showed very strong cytotoxicity against multiple cancer cell lines. 152 mg of the organic extract was fractionated on a Chromabond SPE C18 cartridge. The elution with a step gradient MeOH:H_2_O mixture (0% to 100%) afforded 5 fractions. Anticancer activity was tracked to fraction 3 (F3 44 mg), which contained tsitsikammamines, discorhabdins and epinardins. 40 mg of F3 was further subjected to semi-prep. RP-HPLC chromatography equipped with Onyx Monolithic C18 column using H_2_O/MeCN as the mobile phase. The sub-fractions were collected by their UV absorption at 254 nm (every 1 min if no UV absorption was detected) to yield 33 sub-fractions (F3-1 to F3-33). The tsitsikammamines were tracked in F3-08 (1.3 mg) that was further purified by HPLC coupled to analytic SeQuant^®^ ZIC-HILIC column eluted with mobile phase 20 mM NH_4_CHOO water solution (A) and MeCN (B) to yield compound **1** (0.2 mg, *t*_R_ 3.9 min) and **2** (0.1 mg, *t*_R_ 3.2 min). Discorhabdins and epinardins were tracked in sub-fraction 9 (2.4 mg). Although this sub-fraction had a slightly higher amount, it was very rich in many discorhabdins and epinardins, hence, it was not worked up.

*Tsitsikammamine A* (**1**): Greenish film; ^1^H NMR (DMSO-*d*_6_ and CD_3_OD, 600 MHz) and ^13^C NMR (DMSO-*d*_6_, 150 MHz) Table 2 and Table 3; HR-ESIMS found *m*/*z* [M + H]^+^ 304.1110, C_18_H_14_N_3_O_2_ requires 304.1081.

*16,17-Dehydrotsitsikammamine A* (**2**): Greenish film; due to minor amounts available, no UV and IR data were recorded; ^1^H NMR (CD_3_OD, 600 MHz) and ^13^C NMR (CD_3_OD, extracted from the HMBC spectrum 500 and 125 MHz) Table 2 and Table 3; HR-ESIMS found *m*/*z* [M + H]^+^ 302.0911, C_18_H_12_N_3_O_2_ requires 302.0924.

### 4.4. UPLC-QToF-MS/MS Analysis

The crude organic extract was analyzed on an ACQUITY UPLC I-Class System coupled to the Xevo G2-XS QToF Mass Spectrometer (Waters^®^, Milford, MA, USA) equipped with an electrospray ionization (ESI) source operating with a positive polarity at a mass range of *m/z* 50–1600 Da. The 0.1 mg/mL MeOH solution of the organic extract was filtered through a 0.2 μm PTFE syringe filter (Carl Roth, Karlsruhe, Germany) and then injected (injection volume: 1.0 μL) into the system that was equipped with Acquity UPLC HSS T3 column (High Strength Silica C18, 1.8 µm, 100 × 2.1 mm I.D., Waters^®^) operating at 40 °C. Separation was achieved with a binary LC solvent system controlled by MassLynx^®^ (version 4.1) using mobile phase A 99.9% water/0.1% formic acid (ULC/MS grade) and B 99.9% ACN/0.1% formic acid (ULC/MS grade), pumped at a rate of 0.6 mL/min with the following gradient: initial, 1% B; 0.0–6.0 min to 30% B; 6.0–11.5 min to 100% B; 11.5–13.5 min 100% B, and a column reconditioning phase until 15 min.

ESI conditions were set with the capillary voltage at 0.8 kV, sample cone voltage at 40.0 V, source temperature at 150 °C, desolvation temperature at 550 °C, cone gas flow in 50 L/h and desolvation gas flow in 1200 L/h. MS/MS setting was a ramp collision energy (CE): low CE from 6 eV to 60 eV and high CE from 9 eV to 80 eV. As a control, solvent (methanol) was injected. MassLynx^®^ (Waters^®^, V4.1) was used to analyze the achieved MS and MS^2^ data.

### 4.5. Molecular Networking

The network was created using the UPLC-HRMS/MS data generated from the crude extract of *L. biformis*. All raw MS/MS data were converted from files (.raw) to mzXML file format using MSConvert (version 3.0.10051, Vanderbilt University, USA). The converted data files were uploaded to the Global Natural Products Social molecular networking (http://gnps.ucsd.edu) platform using FileZilla (https://filezilla-project.org/) and a molecular network was generated while using the online workflow [37]. The data were filtered by removing all MS/MS peaks within ±17 Da of the precursor *m/z*. MS/MS spectra were window filtered by choosing only the top 6 peaks in the ±50 Da window throughout the spectrum. The data was then clustered with MS-Cluster with a parent mass tolerance of 0.1 Da and a MS/MS fragment ion tolerance of 0.05 Da to create consensus spectra. Further, consensus spectra that contained less than one spectrum were discarded. A network was then created where edges were filtered to have a cosine score above 0.65 and more than four matched peaks. Further edges between two nodes were kept in the network if and only if each of the nodes appeared in each other′s respective top eight most similar nodes. The spectra in the network were then searched against GNPS spectral libraries. The library spectra were filtered in the same manner as the input data. All of the matches kept between network spectra and library spectra were required to have a score above 0.7 and at least six matched peaks [37]. The output molecular networking data were analysed and visualized using Cytoscape (ver. 3.4.0) [56].

### 4.6. Cytotoxicity Assay

Crude extract of *L. biformis* and downstream fractions were tested in vitro at a final concentration of 200 µg/mL against six human cancer cell lines, Hep G2 (liver cancer cell line, DSMZ, Braunschweig, Germany), HT29 (colorectal adenocarcinoma cell line, DSMZ, Braunschweig, Germany), A375 (malignant melanoma cell line, CLS, Eppelheim, Germany), HCT116 (colon cancer cell line, DSMZ, Braunschweig, Germany), A549 (lung carcinoma cell line, CLS, Eppelheim, Germany), MDA-MB231 (human breast cancer line, CLS, Eppelheim, Germany) and the non-cancerous human keratinocyte cell line HaCaT (CLS, Eppelheim, Germany). By monitoring the metabolic activity at 37 °C under a humidified atmosphere and 5% CO_2_ in CellTiterBlue Cell Viability Assay (Promega, Mannheim, Germany), the sensitivity of the cell lines to the crude extract or fractions was evaluated. Cells were maintained in RPMI 1640 medium (Life Technologies, Darmstadt, Germany) with 10% fetal bovine serum, 100 U/mL penicillin and 100 mg/mL streptomycin supplemented at 37 °C and 5% CO_2_. The cells were seeded in 96 well plates at a concentration of 10,000 cells per well for bioactivity tests. A stock solution of 40 mg/mL in DMSO was prepared for each extract. After 24 h incubation, the medium in the cells was replaced by 100 µL fresh medium containing the test samples. All of the samples were prepared in duplicates. Doxorubicin was used as positive control, while 0.5% DMSO and growth media served as negative controls. After addition of the crude extracts, cells were incubated for 24 h at 37 °C. The assay was performed according to the manufacturer’s instructions (Promega). Cells were incubated for 2 h at 37 °C. Fluorescence at an excitation wavelength of 560 nm and emission at 590 nm was measured using the microplate reader Infinite M200 (Tecan, Crailshaim, Germany). For the determination of IC_50_ values, a dilution series of the extracts or fractions were tested while following the same procedure as described before. IC_50_ values were calculated by using Excel to determine the concentration that show 50% inhibition of the viability.

### 4.7. Molecular Modeling and Docking

Molecular modeling was performed on a DELL Precision T3610 four core workstation using Schrödinger Maestro, version 11.3, 2017, Schrödinger, LLC, New York, NY, USA. The following RCSB protein data bank (pdb) crystal structures were used for modeling studies: 1T8I, 3QX3, 5EK2, 5XE1, 6AZV, 6AZW. Each protein structure was initially prepared by standard settings of the Protein Preparation Wizard 2015-4 (Epik version 2.4, Schrödinger, LLC, 2015; Impact version 5.9, Schrödinger, LLC, 2015; Prime version 3.2, Schrödinger LLC, 2015). For energy minimizations of the small molecule ligands MacroModel, version 11.0, Schrödinger, LLC, 2015 was used. Ionization states and tautomers were generated with LigPrep, version 3.6, Schrödinger, LLC, 2015. Ligand docking and receptor grid generation was performed with Glide, version 6.9, Schrödinger, LLC, 2015. Figures and ligand interaction diagrams (LID) were generated by Maestro.

## Figures and Tables

**Table 1:** Anticancer activity of *Latrunculia biformis* crude extract and fraction 3 that yielded tsitsikammamines. The IC_50_ values are in µg/mL. Positive control doxorubicine.

Sample	A-375	HCT-116	A-549	MB-231	Hep G2	HT-29	HaCaT
Crude extract	2.5	0.8	6.3	3.3	2.4	2.3	3.0
Fraction 3	4.3	1.2	5.0	5.6	5.1	5.2	6.5
Positive control	0.13	10.6	31.4	15.2	14.6	3.0	-

**Table 2:** ^1^H NMR data of compounds **1** (DMSO-*d*_6_ and CD_3_OD) and **2** (CD_3_OD) (600 MHz, *δ* in ppm).

Position	1 ^a^			1 ^b^	2 ^b^
	*δ*_H_^c^, Mult. (*J* in Hz)	*δ*_H_^d^, Mult. (*J* in Hz)	*δ*_H_^e^, Mult. (*J* in Hz)	*δ*_H_^c^, Mult. (*J* in Hz)	*δ*_H_^c^, Mult. (*J* in Hz)
1	7.71 d (8.5)	6.87 d (8.4)	7.36 d (8.5)	7.50 d (8.5)	7.84 d (8.4)
2	6.70 d (8.5)	7.37 d (8.4)	6.86 d (8.5)	6.85 d (8.5)	6.85 d (8.4)
3					
4	6.70 d (8.5)	7.37 d (8.4)	6.86 d (8.5)	6.85 d (8.5)	6.85 d (8.4)
5	7.71 d (8.5)	6.87 d (8.4)	7.36 d (8.5)	7.50 d (8.5)	7.84 d (8.4)
6					
7					
8	7.14 d (1.8)	7.16 d (2.5)	7.17 d (2.9)	7.02 s	7.22 s
NH-9	12.28 s	13.28 br s	13.32 s		
10					
11					
12					
NH-13	12.00 s	13.01 br s	13.04 s		
14	6.91 s	7.10 d (1.8)	7.12 d (2.6)	6.91 s	7.93 s
15					
16	2.65 t (7.8)	2.93 t (7.8)	2.93 t (7.8)	2.89 t (7.8)	7.66 d (5.9)
17	3.97 t (7.8)	3.85 t (7.8)	3.84 t (7.8)	3.94 t (7.8)	8.34 d (5.9)
19					
20					
21					
OH	9.31 br s	10.60 br s	10.43 s		

^a^ DMSO-*d*_6_; ^b^ CD_3_OD; ^c^ free base; ^d^ data from literature [30]; ^e^ TFA salt.

**Table 3:** ^13^C NMR data of compounds **1** (DMSO-*d*_6_) and **2** (CD_3_OD) (150 MHz, *δ* in ppm).

Position	1 ^a^			2 ^b,c^
	*δ*_C_^d^, Type	*δ*_C_^e^, Type	*δ*_C_^f^, Type	*δ*_C_^d^, Type
1	129.8, CH	116.2, CH	129.1, CH	129.8, CH
2	114.4, CH	128.9, CH	116.4, CH	114.4, CH
3	156.1, C	157.6, C	157.7, C	156.1, C
4	114.4, CH	128.9, CH	116.4, CH	114.4, CH
5	129.8, CH	116.2, CH	129.1, CH	129.8, CH
6	125.1, C	127.2, C	127.5, C	126.1, C
7	124.3, C	122.4, C	122.5, C	124.9, C
8	122.8, CH	125.0, CH	125.2, CH	123.1, CH
10	133.2, C	134.6, C	134.8, C	134.5, C
11	167.7, C	166.3, C	166.5, C	n.o.
12	124.7, C	127.8, C	128.0, C	125.2, C
14	121.2, CH	123.1, CH	123.3, CH	123.9, CH
15	117.4, C	119.2, C	119.4, C	124.8, C
16	17.9, CH_2_	17.6, CH_2_	17.8, CH_2_	114.0, CH
17	49.7, CH_2_	45.0, CH_2_	45.0, CH_2_	140.6, CH
19	154.1, C	157.6, C	157.0, C	146.7, C
20	121.6, C	113.5, C	113.5, C	122.7, C
21	121.8, C	120.7, C	120.9, C	120.7, C

^a^ DMSO-*d*_6_; ^b^ CD_3_OD; ^c^ Retrieved from HSQC and HMBC spectra; ^d^ free base; ^e^ data from literature [30]; ^f^ TFA salt; n.o: not observed.

## Supplementary Materials

The following are available online at http://www.mdpi.com/1660-3397/16/8/268/s1, HR-ESIMS and NMR spectra of compounds **1** and **2**. HR-ESIMS and NMR spectra of compounds **1** and **2**, and in vitro anticancer activity data of the *Latrunculia* extract and its SPE fractions.
