# Supplementary material for: Targeted Isolation of Tsitsikammamines from the Antarctic Deep-Sea Sponge Latrunculia biformis by Molecular Networking and Anticancer Activity
Source: Mar Drugs. 2018 Aug 2;16(8):268. doi: 10.3390/md16080268 (PMC6117724; doi:10.3390/md16080268)
Supplement: Supplementary file 1 [file marinedrugs-16-00268-s001.pdf]

SUPPLEMENTARY MATERIALS

Targeted Isolation of Tsitsikammamines from the Antarctic Deep-Sea Sponge *Latrunculia biformis* by Molecular Networking and Anticancer Activity

Fengjie Li [1], Dorte Janussen [2], Christian Peifer [3], Ignacio Pérez-Victoria [4] and Deniz Tasdemir [1,5,*]

1. GEOMAR Centre for Marine Biotechnology (GEOMAR-Biotech), Marine Natural Products Research Unit Chemistry, GEOMAR Helmholtz Centre for Ocean Research Kiel, Am Kiel-Kanal 44, 24106 Kiel, Germany; fli@geomar.de
2. Senckenberg Research Institute and Natural History Museum, Senckenberganlage 25, 60325 Frankfurt, Germany; dorte.janussen@senckenberg.de
3. Pharmaceutical Chemistry, Kiel University, Gutenbergstraße 76, 24118 Kiel, Germany; cpeifer@pharmazie.uni-kiel.de
4. Fundación MEDINA, Parque Tecnológico de la Salud, Av. Conocimiento 18016 Granada, Spain; ignacio.perez-victoria@medinaandalucia.es
5. Faculty of Mathematics and Natural Sciences, Kiel University, Christian-Albrechts-Platz 4, 24118 Kiel, Germany

* Correspondence: dtasdemir@geomar.de; Tel.: +49-431-600-4430

**Figure No**	**Page No**
**Figure S1.** ^{1}H NMR spectrum of compound **1** (free base, 600 MHz, DMSO-*d*6).	3
**Figure S2.** ^{13}C NMR spectrum of compound **1** (free base, 150 MHz, DMSO-*d*6).	3
**Figure S3.** HSQC NMR spectrum of compound **1** (free base, 600 MHz, DMSO-*d*6).	4
**Figure S4.** HMBC spectrum of compound **1** (free base, 600 MHz, DMSO-*d*6).	4
**Figure S5.** COSY spectrum of compound **1** (free base, 600 MHz, DMSO-*d*6).	5
**Figure S6.** NOESY spectrum of compound **1** (free base, 600 MHz, DMSO-*d*6).	5
**Figure S7.** ^{1}H NMR spectrum of compound **1** (free base, 600 MHz, MeOD).	6
**Figure S8.** ^{1}H NMR spectrum of compound **1** (TFA salt, 600 MHz, DMSO-*d*6).	6
**Figure S9.** ^{13}C NMR spectrum of compound **1** (TFA salt, 150 MHz, DMSO-*d*6).	7
**Figure S10.** HR-ESIMS spectrum of compound **1**.	7
**Figure S11.** ^{1}H NMR spectrum of compound **2** (600 MHz, MeOD).	8
**Figure S12.** HSQC spectrum of compound **2** (600 MHz, MeOD).	8
**Figure S13.** HMBC spectrum of compound **2** (600 MHz, MeOD).	9
**Figure S14.** HMBC spectrum of compound **2** (500 MHz, MeOD).	9
**Figure S15**. COSY spectrum of compound **2** (500 MHz, MeOD).	10
**Figure S16.** NOESY spectrum of compound **2** (500 MHz, MeOD).	10
**Figure S17.** HR-ESIMS spectrum of compound **2**.	11
**Figure S18.** In vitro activity of crude *Latrunculia* extract and its SPE fractions against cancer cell lines.	11

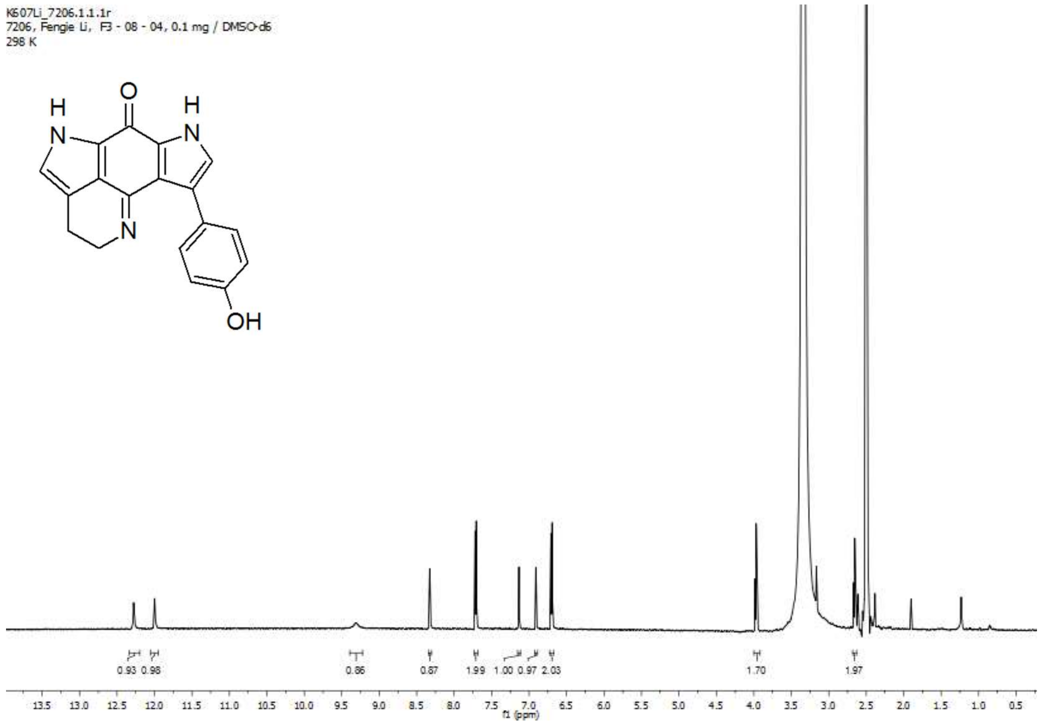

Figure S1. ^{1}H NMR spectrum of compound **1** (free base, 600 MHz, DMSO-*d*6).

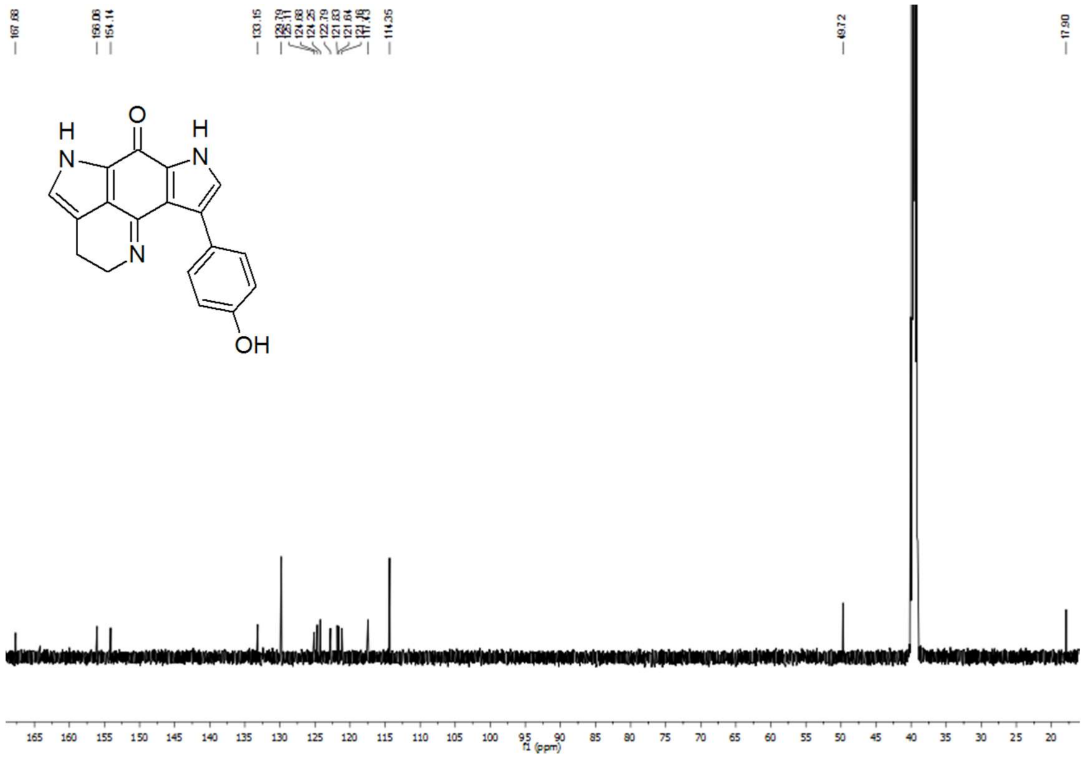

Figure S2. ^{13}C NMR spectrum of compound **1** (free base, 150 MHz, DMSO-*d*6).

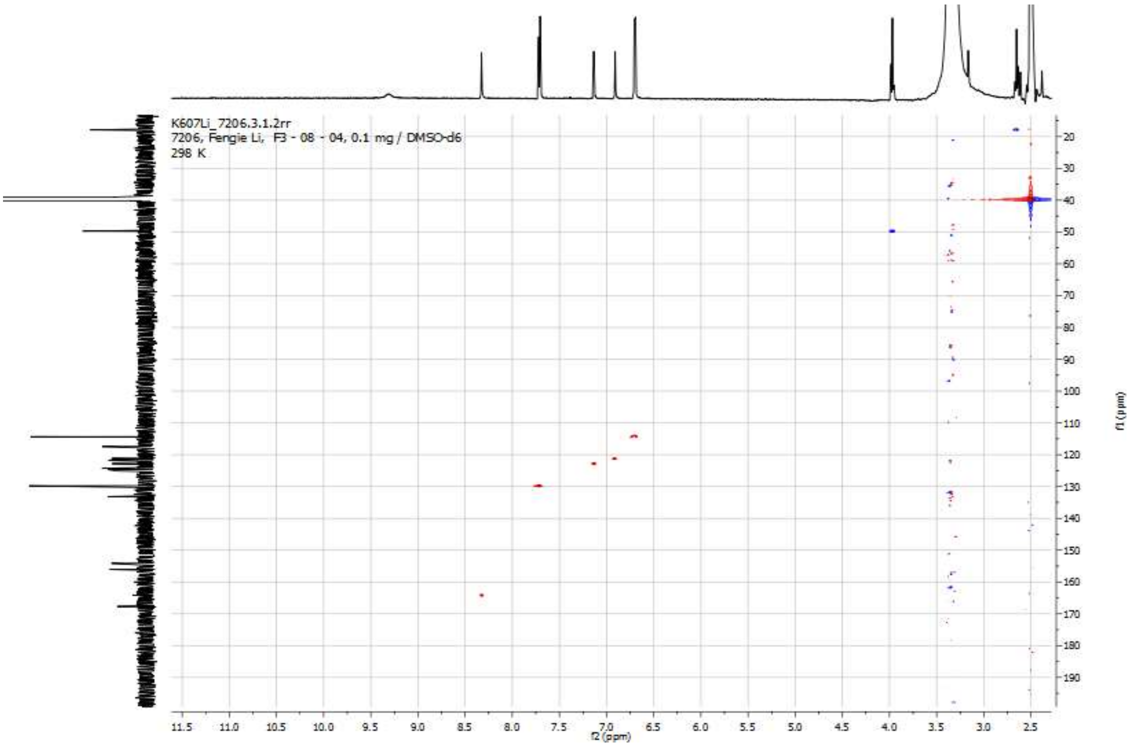

Figure S3. HSQC NMR spectrum of compound **1** (free base, 600 MHz, DMSO-*d*6).

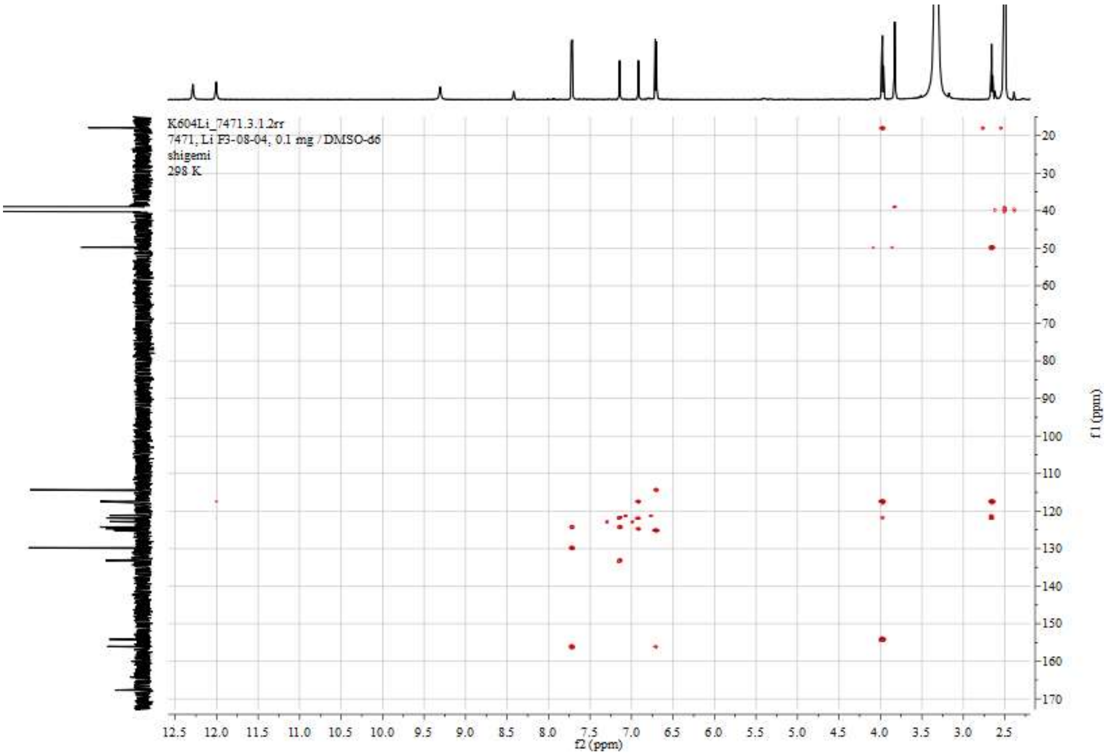

Figure S4. HMBC spectrum of compound **1** (free base, 600 MHz, DMSO-*d*6).

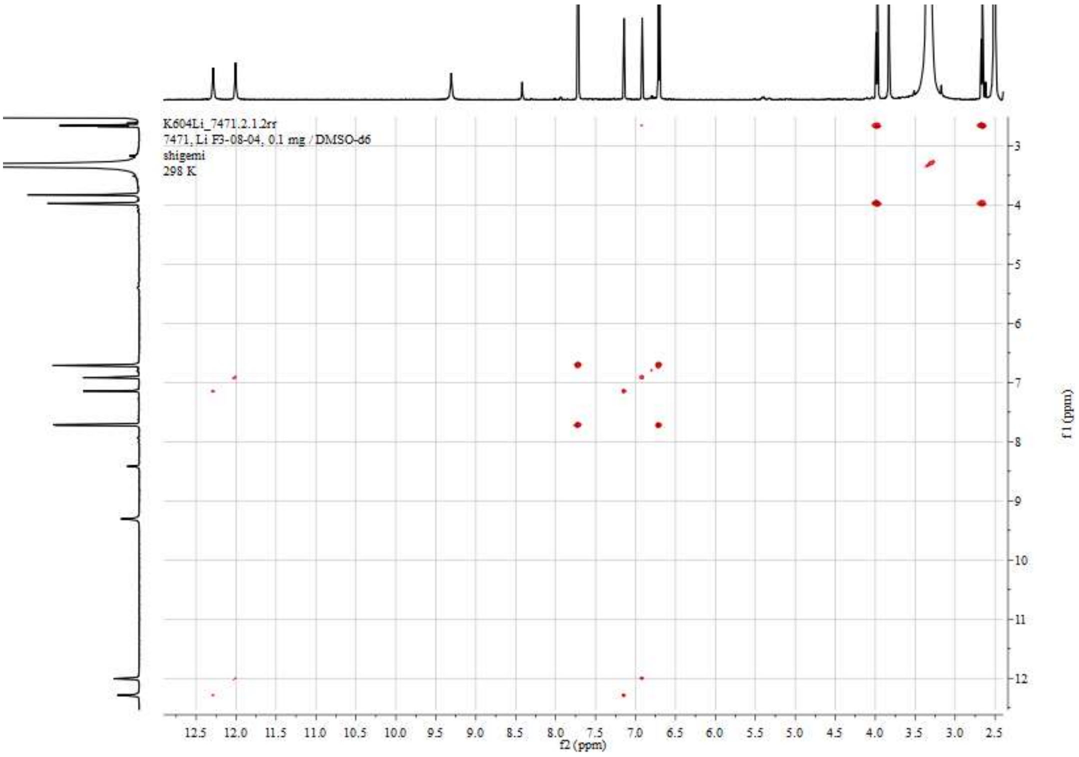

Figure S5. COSY spectrum of compound **1** (free base, 600 MHz, DMSO-*d*6).

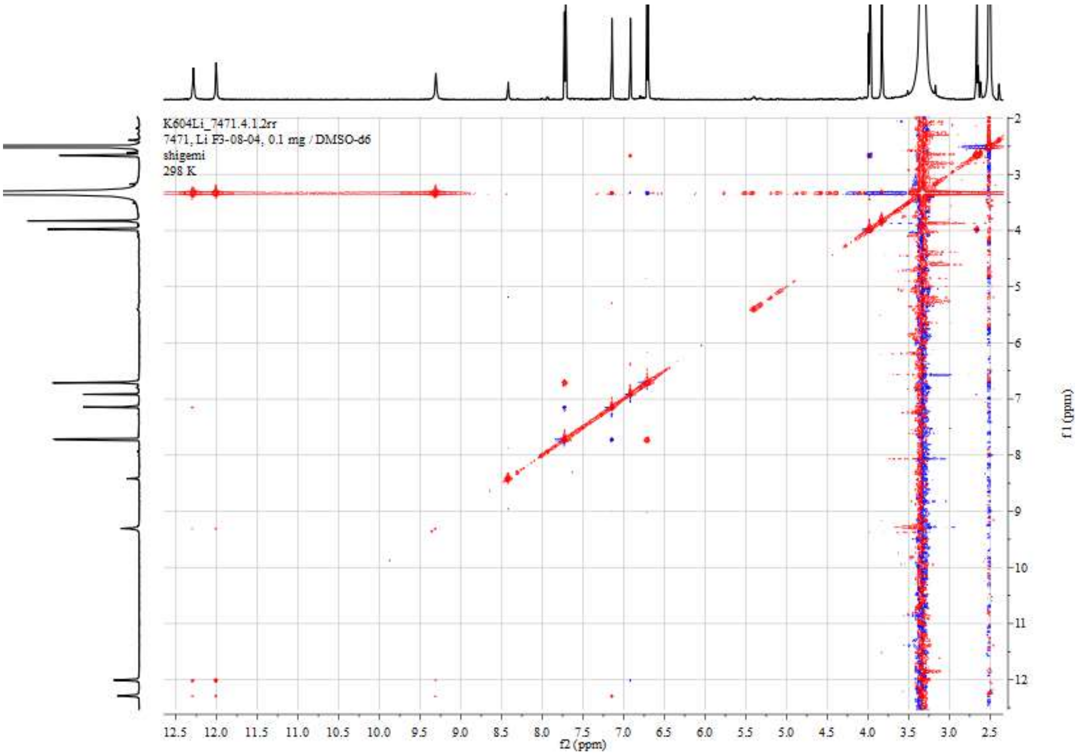

Figure S6. NOESY spectrum of compound **1** (free base, 600 MHz, DMSO-*d*6).

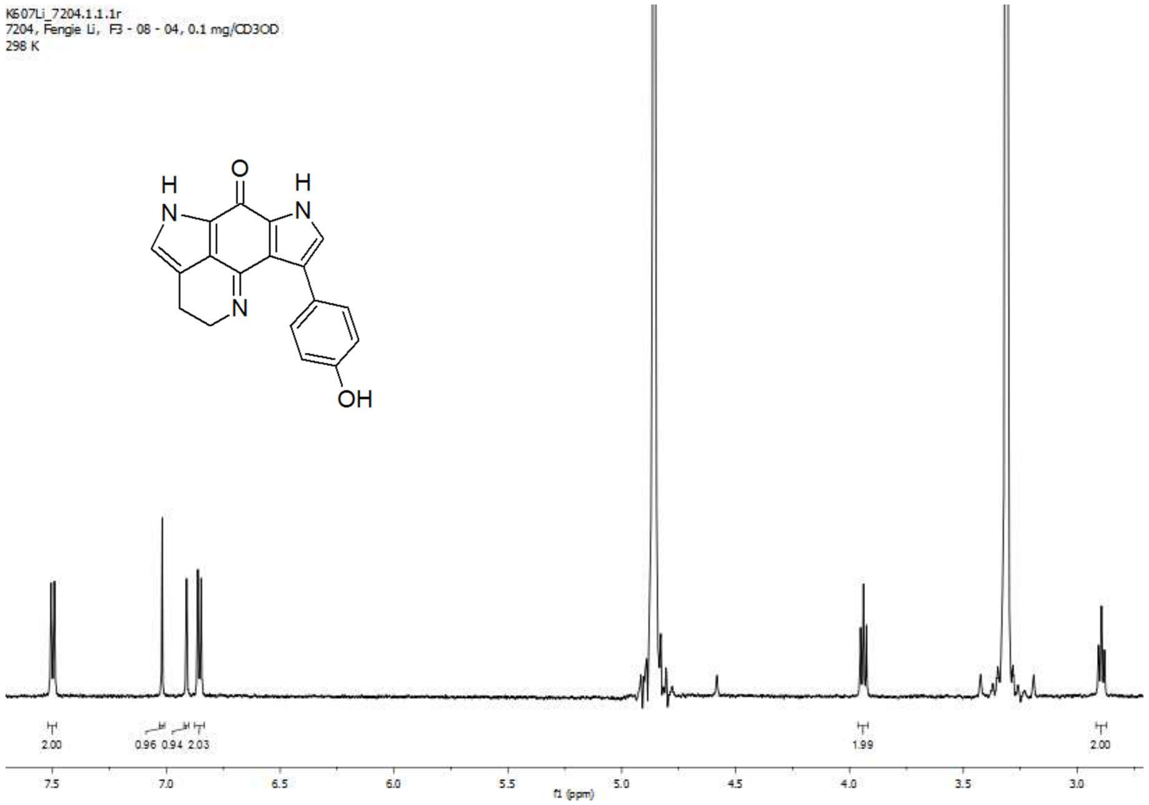

Figure S7. ^{1}H NMR spectrum of compound **1** (free base, 600 MHz, MeOD).

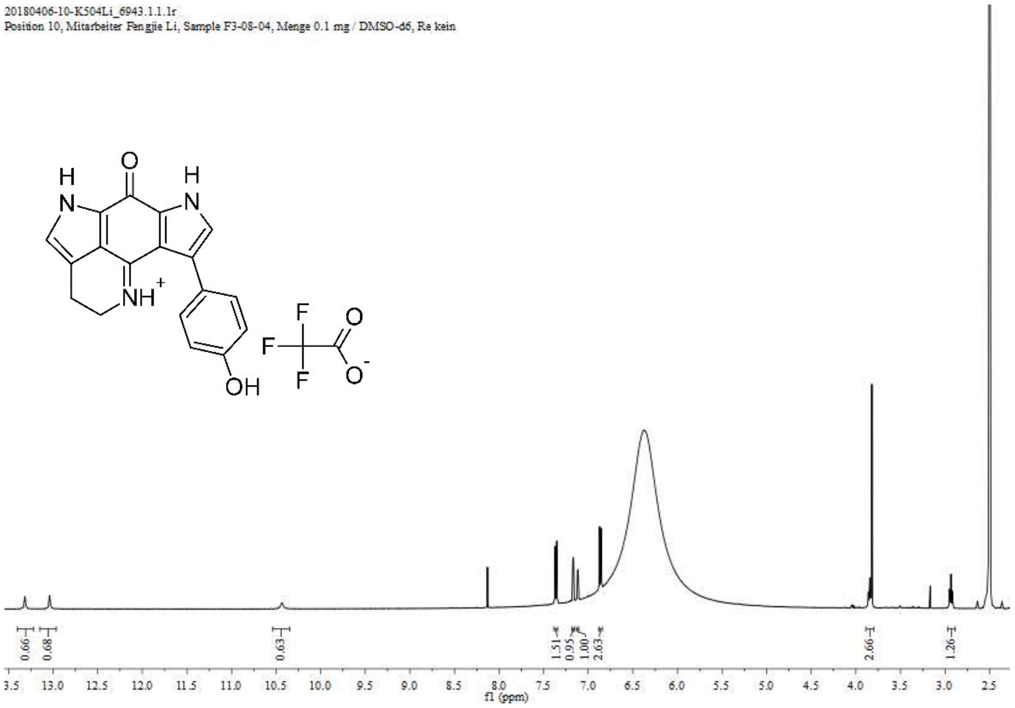

Figure S8. ^{1}H NMR spectrum of compound **1** (TFA salt, 600 MHz, DMSO-*d*6).

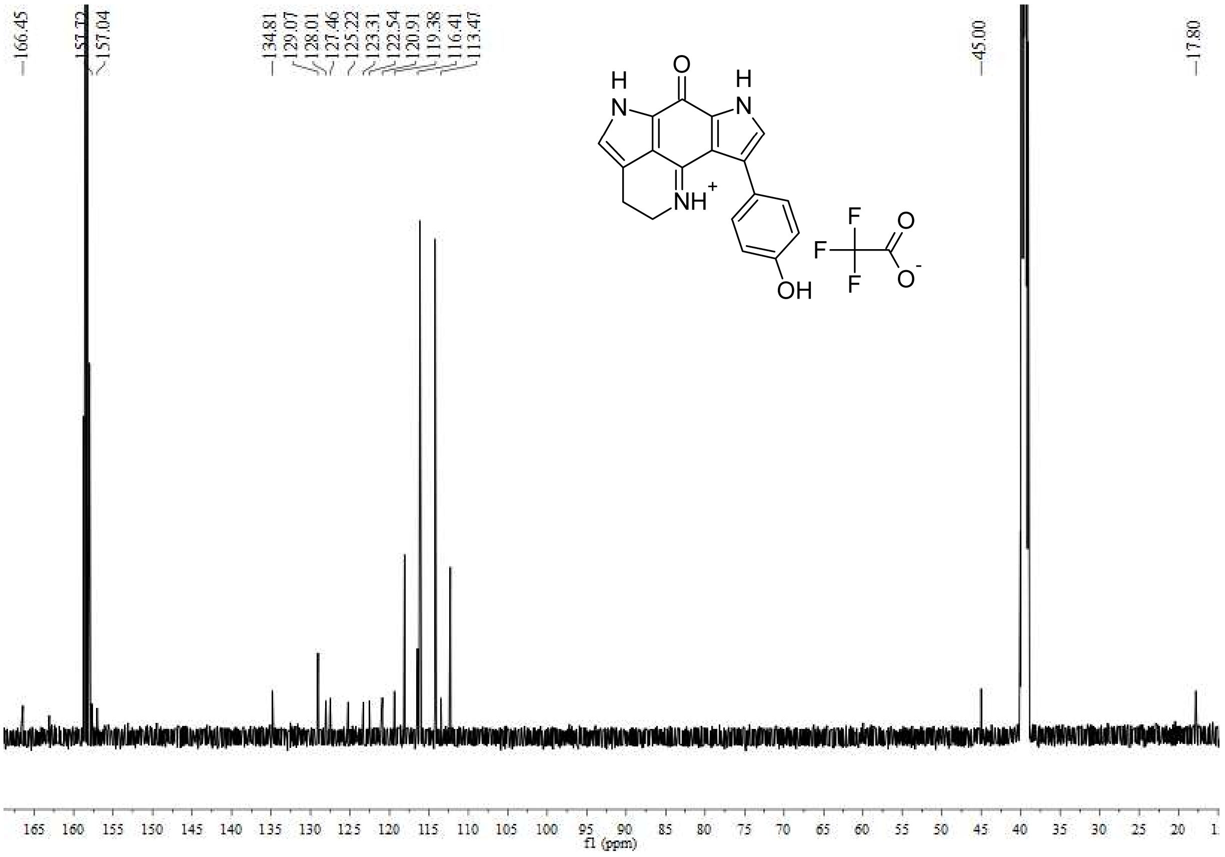

Figure S9. ^{13}C NMR spectrum of compound **1** (TFA salt, 150 MHz, DMSO-*d*6).

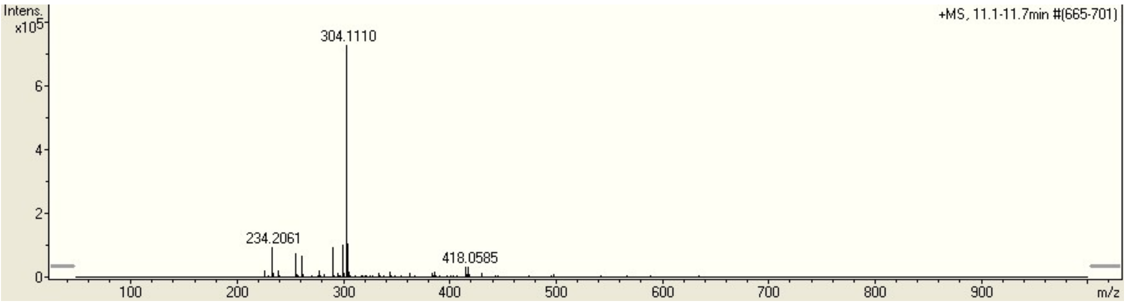

Figure S10. HR-ESIMS spectrum of compound **1**.

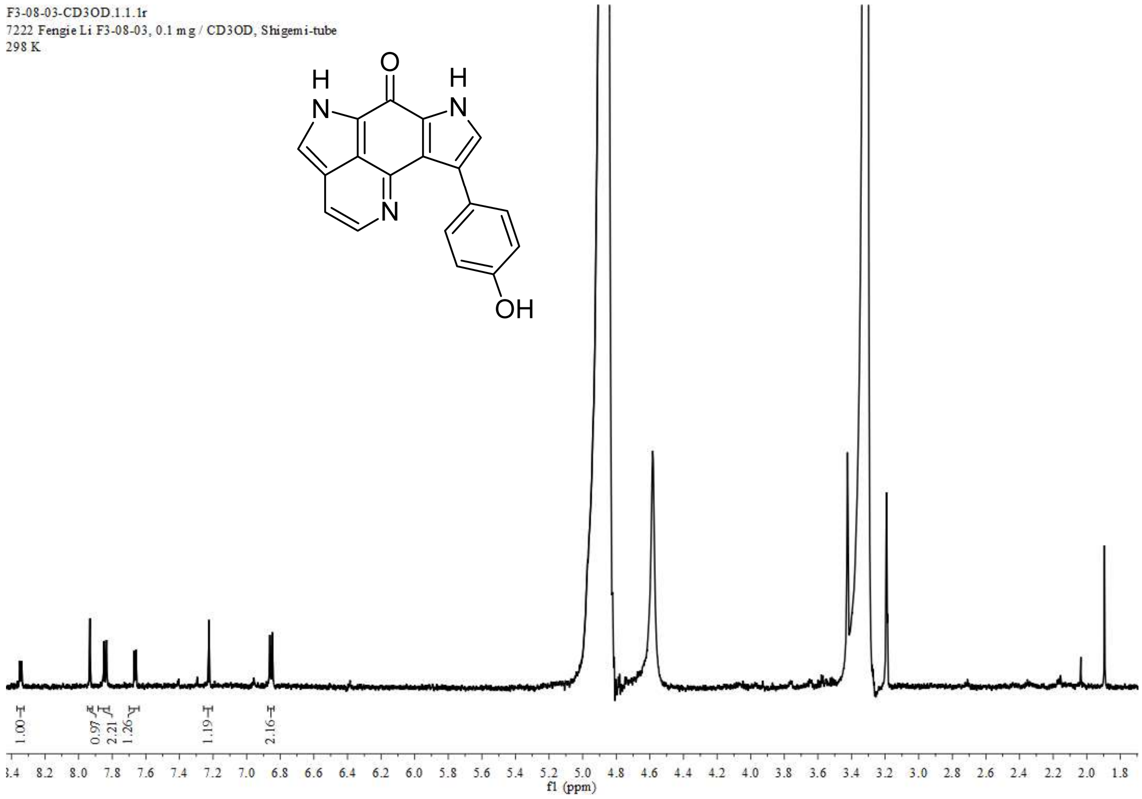

Figure S11. ^{1}H NMR spectrum of compound **2** (600 MHz, MeOD).

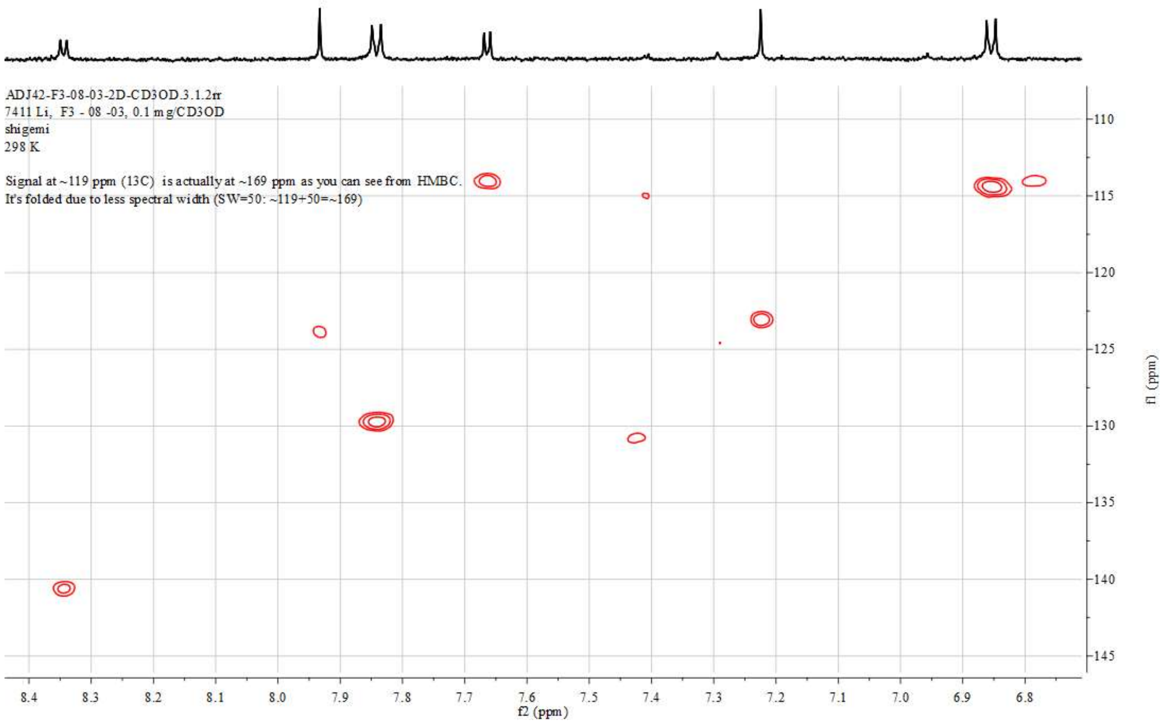

Figure S12. HSQC spectrum of compound **2** (600 MHz, MeOD).

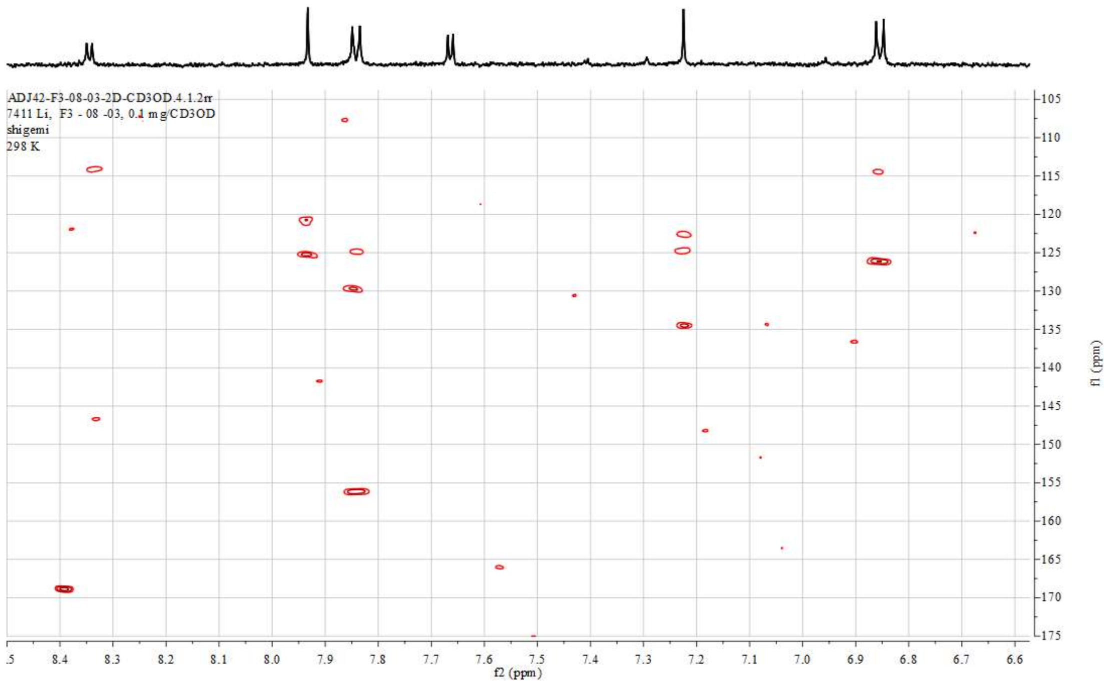

Figure S13. HMBC spectrum of compound **2** (600 MHz, MeOD).

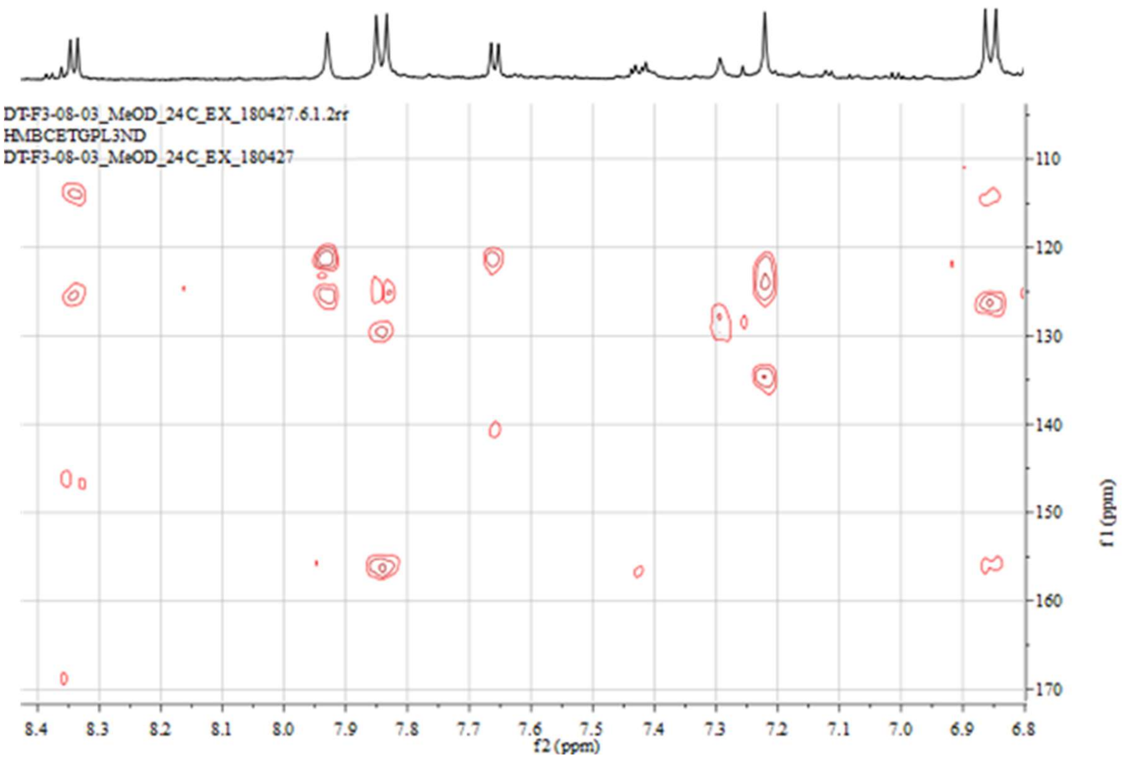

Figure S14. HMBC spectrum of compound **2** (500 MHz, MeOD).

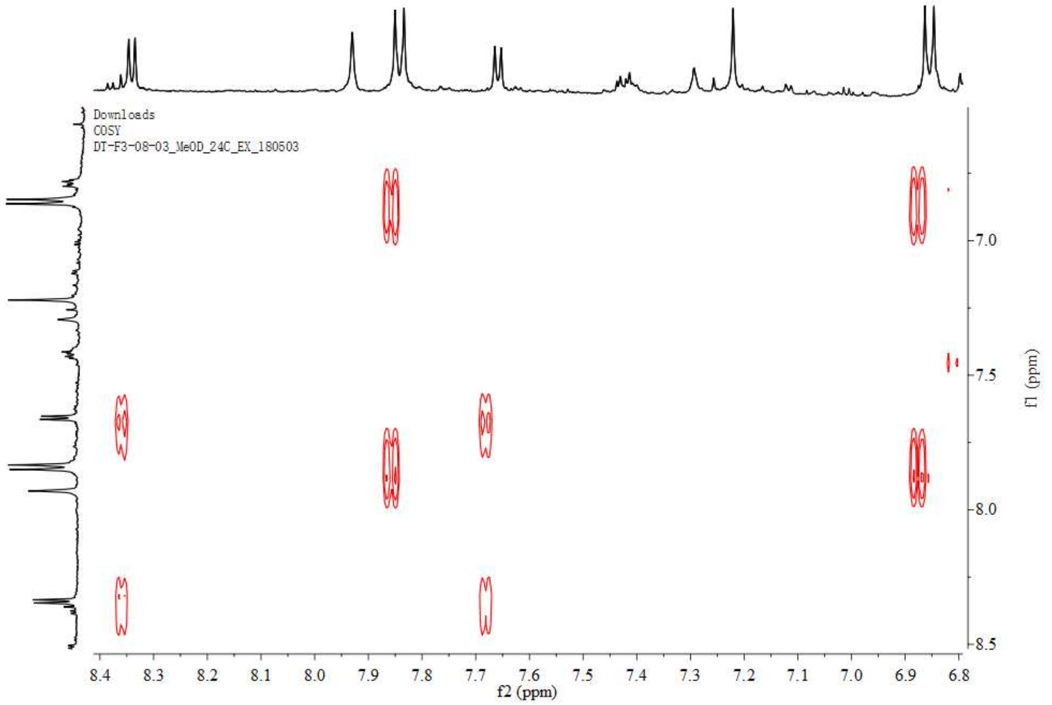

Figure S15. COSY spectrum of compound **2** (500 MHz, MeOD).

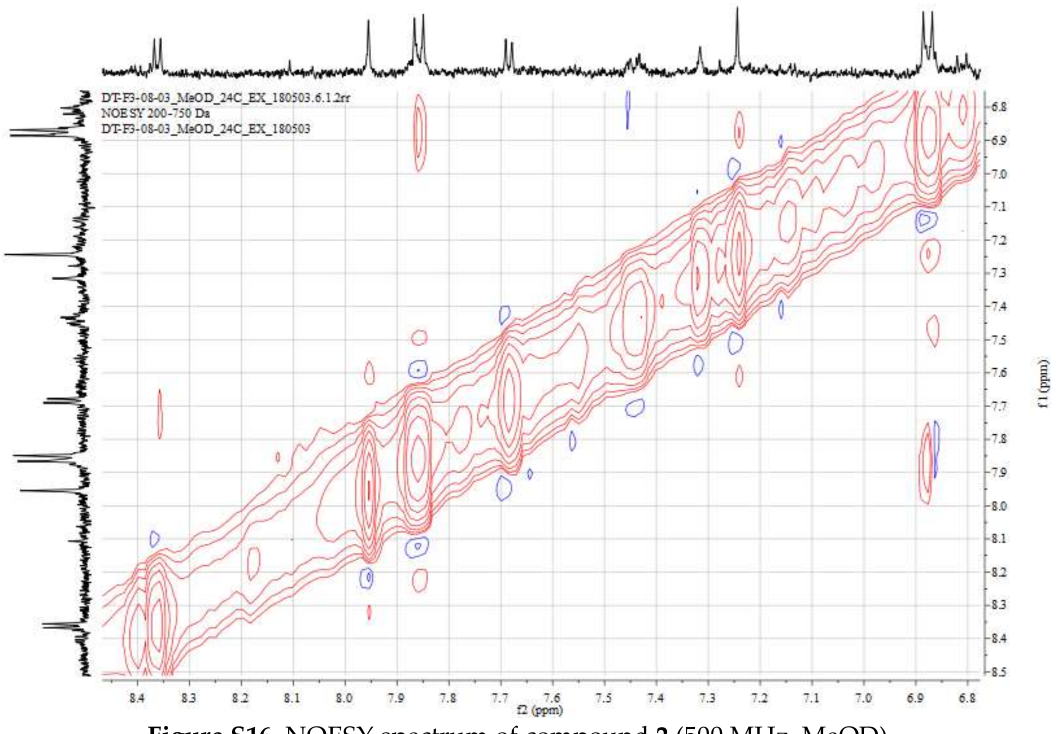

Figure S16. NOESY spectrum of compound **2** (500 MHz, MeOD).

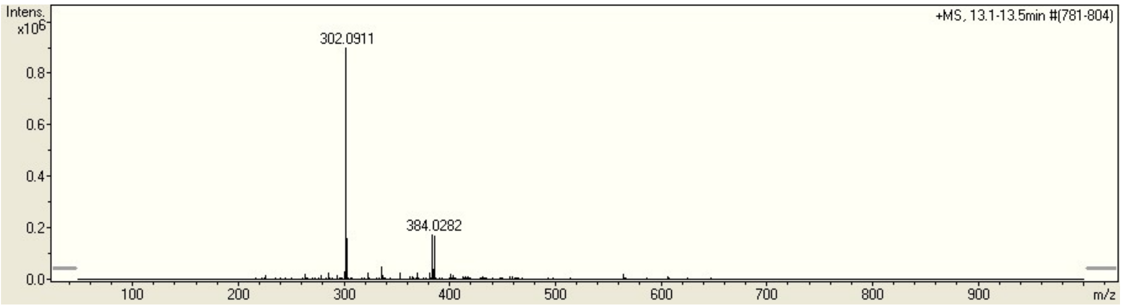

Figure S17. HR-ESIMS spectrum of compound **2**.

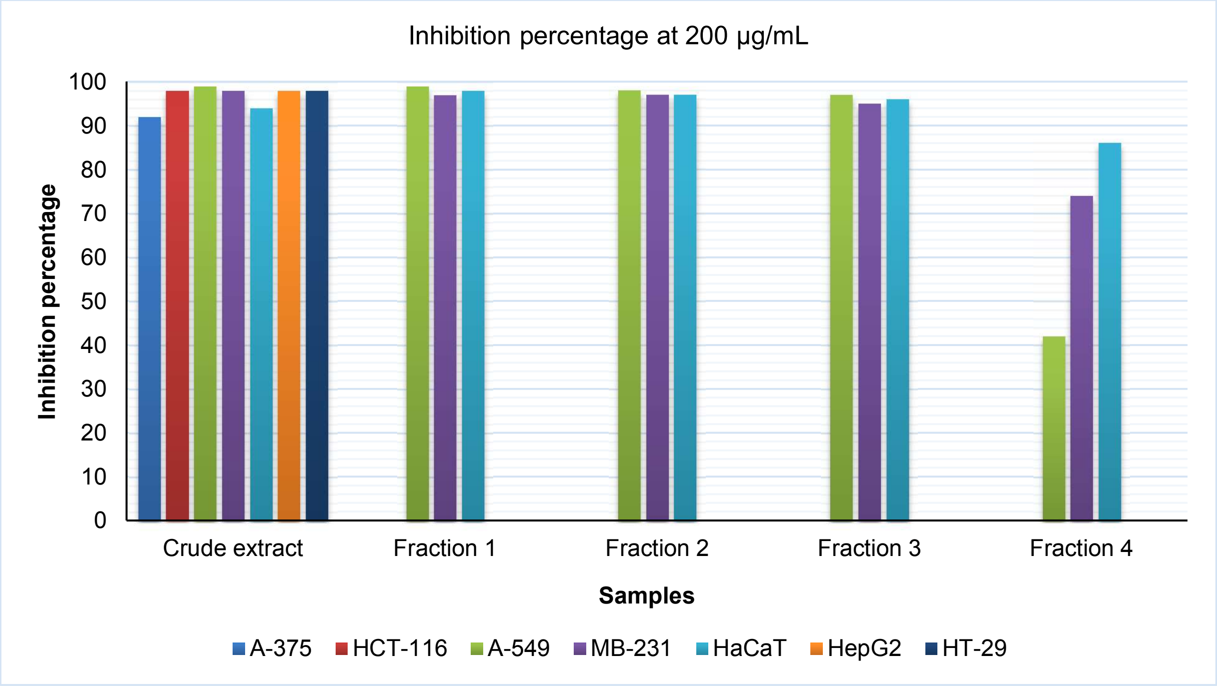

Figure S18. In vitro activity of crude *Latrunculia* extract and its SPE fractions against cancer cell lines. Test concentration: 200 µg/mL. Because of limited amounts available, fraction 5 was not tested against any cell lines while the other 4 fractions were tested only against three cancer cell lines.
